# Nivolumab Induced Reactivation of Hepatitis B in a Patient with Metastatic Gastric Adenocarcinoma—A Case Report

**DOI:** 10.3390/reports9030256

**Published:** 2026-08-06

**Authors:** Jan Naseer Kaur, Parikshit Padhi, Abhinav Dodeja

**Affiliations:** 1 Department of Microbiology and Immunology, College of Osteopathic Medicine, D’Youville University, Buffalo, NY 14201, USA; 2 Department of Internal Medicine, Jacobs School of Medicine and Biomedical Sciences, University at Buffalo, Buffalo, NY 14203, USA; 3 Department of Internal Medicine, Baystate Health, Springfield, MA 01199, USA

**Keywords:** Nivolumab, Hepatitis B, reactivation, gastric cancer

## Abstract

**Background and Clinical Significance:** The most common cause of liver toxicity with the use of immune checkpoint inhibitors (ICIs) is autoimmune hepatitis. As most patients with prior viral infections such as hepatitis B and hepatitis C were excluded in trials for the use of ICIs, the safety of ICIs in these patients with active or prior treated hepatitis is unknown. With expanded use of these medications in many malignancies, it is important to understand the risk of viral reactivation with these medications. There are only few case series and reports documenting hepatitis B reactivations with the use of ICIs. **Case Presentation:** We present a middle-aged woman with a history of treated hepatitis B who presented with metastatic gastric cancer. She was treated with two cycles of 5-FU, oxaliplatin and nivolumab followed by maintenance nivolumab. After 14 months of nivolumab, she developed marked transaminitis and was found to have reactivation of hepatitis B. As autoimmune hepatitis was the initial suspicion, the patient was initiated on prednisone 1 mg/kg with no improvement in transaminases. Due to the significant elevation of HBV DNA, she was diagnosed with hepatitis B reactivation. She was initiated on entecavir with normalization of transaminases and improvement in HBV DNA levels. She was successfully rechallenged with nivolumab with no evidence of recurrent transaminitis or worsening HBV DNA levels. **Conclusions:** There are case series of HBV reactivation with the use of ICIs. We believe that any patients with known history of HBV should get baseline viral titers prior to initiation of ICIs with serial monitoring of DNA levels. Prospective studies to evaluate risk of reactivation may need to be performed for us to get a better understanding of risks of viral reactivation and potential effects it may have on safety and efficacy of ICIs.

## 1. Introduction and Clinical Significance

Immune checkpoint inhibitors (ICIs) have transformed the landscape of cancer treatment by targeting immune regulatory pathways, such as the programmed cell death protein-1 (PD1) and programmed death-ligand 1 (PDL1) axis, as well as the cytotoxic T-lymphocyte antigen-4 (CTLA-4) pathway. These therapies block inhibitory signals that suppress the immune system, thereby enhancing the body’s ability to mount an antitumor response. Nivolumab, an anti-PD1 monoclonal antibody, works by inhibiting the PD1 signaling pathway, which in turn restores T-cell-mediated antitumor activity. Initially approved by the FDA in 2014, the indications for nivolumab have rapidly expanded across a broad spectrum of advanced cancers [1].

Despite the significant advancements in cancer therapy, there is limited evidence regarding the safety and efficacy of ICIs in patients with chronic hepatitis B virus (HBV) infection. HBV remains a major global health burden, even though it can be effectively prevented through vaccination. The global prevalence of HBV is particularly concerning in patients with cirrhosis, where it reaches around 42% [2]. The virus is significantly more prevalent in resource-limited regions, such as parts of Asia and Africa, compared to North America and Europe [3].

Data on HBV reactivation in patients receiving nivolumab is scarce. Although case reports and studies on other ICIs have been published, reports specifically detailing the risk of HBV reactivation in patients treated with nivolumab remain limited. This case represents the first documented instance of HBV reactivation in a patient with advanced gastric cancer undergoing nivolumab therapy, highlighting the need for further investigation into the risks and management of HBV in patients receiving ICI therapy.

## 2. Case Presentation

**Figure 1:**
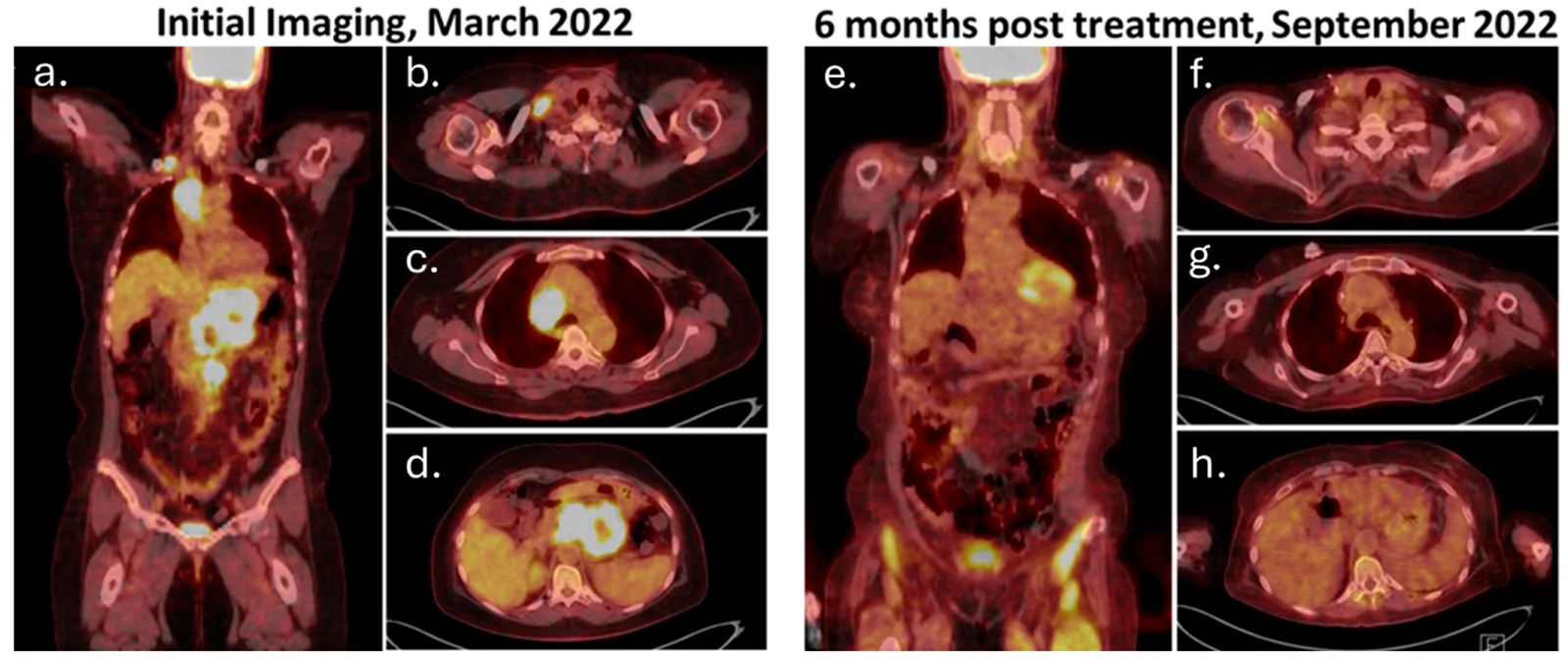
Baseline and post-treatment PET imaging demonstrating complete metabolic response. Initial PET imaging obtained at diagnosis in March 2022 demonstrated FDG-avid primary gastric disease with extensive nodal involvement. (**a**) shows the baseline coronal PET image demonstrating the primary gastric mass with conglomerate gastrohepatic and celiac axis lymphadenopathy measuring 76 mm, as well as FDG-avid adenopathy involving the right neck, right paratracheal, and para-aortic regions. (**b**) shows a right supraclavicular lymph node measuring 11 mm with SUV 7.9. (**c**) shows a right paratracheal lymph node measuring 42 mm with SUV 14.3. (**d**) shows the primary gastric mass with conglomerate gastrohepatic and celiac adenopathy measuring 76 mm with SUV 14.8. Follow-up PET imaging after nivolumab-based therapy is shown in (**e**–**h**) and demonstrates complete metabolic response, with resolution of FDG avidity in the right supraclavicular lymph node, right paratracheal lymph node, and primary gastric mass, along with complete anatomic resolution of the gastric mass. FDG, fluorodeoxyglucose; PET, positron emission tomography; SUV, standardized uptake value.

A 57-year-old female with a history of hypertension and chronic HBV treated in Vietnam in 2013 (unknown course of treatment as we had no records and patient does not remember her treatment history) presented with epigastric abdominal pain radiating to her back. She was diagnosed with Stage IV gastric adenocarcinoma with metastases to the liver and to the lymph nodes in the chest and abdomen. Her adenocarcinoma was noted to be HER2-negative, mismatch repair [MMR]-proficient and PDL1-positive at 80%. She was started on treatment with palliative intent with Fluorouracil (5-FU), Leucovorin and Oxaliplatin (FOLFOX) and nivolumab. Prior to initiating systemic treatment, her hepatitis titers were checked. The hepatitis B surface antigen (HBsAg) and core antibody (HBcAb) were positive and the HBV DNA was 968 units/mL. The hepatitis C panel was negative. Anti-HBs status was not available in the clinical records. Baseline liver function tests were within normal limits. FOLFOX was discontinued after the second cycle due to intolerance and she was continued with single-agent nivolumab (240 mg IV) every 2 weeks. Baseline PET imaging obtained at diagnosis demonstrated extensive FDG-avid disease, including a large primary gastric mass with conglomerate gastrohepatic and celiac axis lymphadenopathy, as well as FDG-avid nodal involvement in the right supraclavicular, right paratracheal, and para-aortic regions. Follow-up PET imaging after nivolumab-based therapy showed complete metabolic response, with resolution of FDG avidity in the previously involved nodal sites and primary gastric lesion (Figure 1). This radiographic response supported continuation of single-agent nivolumab after discontinuation of FOLFOX due to intolerance. Follow-up imaging demonstrated complete metabolic response. As PDL1 was high and due to the positive response, single-agent nivolumab was continued. After 28 cycles of nivolumab, she continued to have no evidence of recurrence.

She was admitted to the hospital after cycle 28 of nivolumab with fevers, chills, and right-upper quadrant abdominal pain. Her liver function tests (LFTs) were noted to be elevated and complete blood count showed mild cytopenias (Table 1). She had denied any alcohol use or significant acetaminophen use. There was a concern for autoimmune hepatitis, and she was initiated on prednisone 1 mg/kg daily with no improvement in LFT after being on steroids for 2 weeks. Given her prior history of HBV infection, we ordered a hepatitis B panel and found a HBV viral load of nearly 7 million IU/mL. Once reactivation of hepatitis B was confirmed, we discontinued prednisone. Regarding her prior treatment for hepatitis B in Vietnam, we had no medical records of her treatment, nor did we have any medical records of her initial viral titers and response to treatment. She was started on entecavir 0.5 mg/day after consultation with hepatology, with improvement in transaminitis within 2 weeks and normalization of LFT in 2 months (see Table 1). The HBV DNA levels also improved to 25,900 IU/mL after 2 weeks of entecavir.

**Figure 2:**
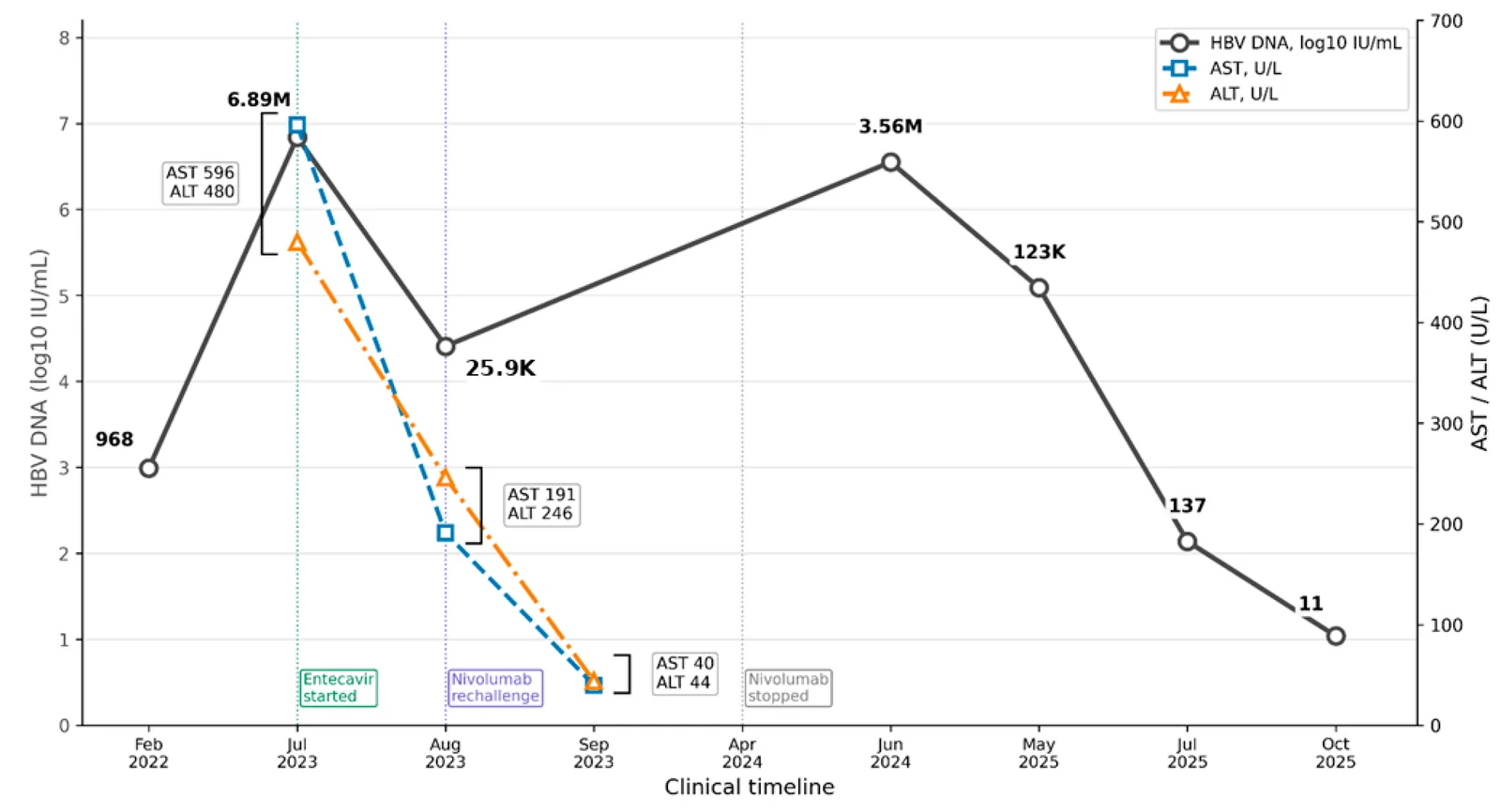
Clinical course of HBV reactivation during nivolumab therapy. HBV DNA PCR viral load is plotted on the left y-axis as log10 IU/mL, with absolute HBV DNA values labeled at each timepoint. AST and ALT are plotted on the right y-axis in U/L, with paired liver enzyme values grouped at each available liver-enzyme timepoint. Key clinical events, including initiation of entecavir, nivolumab rechallenge, and nivolumab discontinuation, are indicated along the timeline. The figure highlights the marked HBV DNA increase and transaminase elevation at the time of hepatitis flare, followed by biochemical improvement and viral suppression after entecavir initiation. ALT, alanine aminotransferase; AST, aspartate aminotransferase; HBV, hepatitis B virus; IU/mL, international units per milliliter; PCR, polymerase chain reaction.

The temporal relationship between nivolumab exposure, HBV DNA elevation, transaminase abnormalities, antiviral initiation, biochemical recovery, and subsequent nivolumab rechallenge is summarized in Figure 2. The patient’s HBV DNA increased markedly at the time of hepatitis flare, accompanied by significant AST and ALT elevation. Following initiation of entecavir, liver enzymes improved rapidly and normalized within approximately two months, while HBV DNA levels declined substantially. This clinical course supported HBV reactivation, rather than immune-mediated hepatitis alone, as the primary driver of liver injury. She was restarted on nivolumab after a 2-month break with no worsening of her transaminitis or any evidence of recurrence of cancer 2 years after initiation of nivolumab. Due to the stability of her disease, treatment was discontinued after 2 years of nivolumab (received a total of 48 cycles) with plans for surveillance. Three months after discontinuing nivolumab, there was no evidence of recurrence of her cancer. Unfortunately in April 2024 (8 months after initiation of entecavir), she lost insurance approval and had to be off treatment for 6 weeks. During that time the HBV viral load rose to 3,560,000 IU/mL. At that time she was asymptomatic and denied any abdominal symptoms or jaundice. This later increase in HBV DNA after interruption of antiviral therapy, despite preserved liver enzyme normalization, further underscores the importance of ongoing HBV-directed antiviral suppression and longitudinal viral load monitoring in patients receiving or recently treated with immune checkpoint inhibitors. She was able to procure the medication in July 2024 and has been on it since with a positive response in the HBV DNA titers. She is currently still following up with her gastroenterologist and despite the rise in HBV viral load, the LFTs are still normal, and she remains on surveillance for metastatic gastric cancer as there is no evidence of recurrent cancer.

## 3. Discussion

In early clinical trials of nivolumab, patients with chronic HBV, HCV, and HIV were largely excluded due to concerns regarding potential viral reactivation and associated toxicity. However, HBV reactivation during ICI therapy has been documented in a limited number of case reports, case series, and retrospective studies [4,5,6,7]. The exact rate of HBV reactivation with ICI therapy varies, depending on factors such as the population studied and prophylactic antiviral use.

Hepatitis B virus persists in hepatocytes as covalently closed circular DNA (cccDNA), which serves as a stable viral reservoir. This cccDNA can persist even after clinical recovery, allowing for long-term viral persistence despite undetectable circulating virus [8]. In chronic hepatitis B infection, HBV-specific T cells exhibit functional exhaustion with increased expression of inhibitory receptors such as PD-1 [9]. The PD-1/PD-L1 pathway has been shown to contribute to impaired antiviral T-cell responses in chronic HBV infection [9]. Blockade of PD-1/PD-L1 engagement has been shown in experimental studies to improve HBV-specific T-cell function [9]. In parallel, clinical reports have documented HBV reactivation in patients receiving immune checkpoint inhibitors such as nivolumab [10].

This case also highlights the diagnostic challenge of distinguishing immune-related hepatitis from HBV reactivation in patients receiving ICIs. Both conditions may present with acute transaminase elevation, and immune-mediated hepatitis is often considered early in the differential diagnosis. However, the marked elevation in HBV DNA, lack of biochemical improvement with corticosteroids, and rapid improvement after initiation of entecavir supported HBV reactivation as the primary etiology of liver injury in our patient. This distinction is clinically important because management differs substantially: immune-mediated hepatitis is typically treated with corticosteroids or other immunosuppressive agents, whereas HBV reactivation requires prompt antiviral therapy.

One retrospective case series of patients with HIV, HBV, or HCV who received ICIs for advanced cancers reported no instances of HBV reactivation. Similarly, a clinical trial of nivolumab in patients with advanced hepatocellular carcinoma also did not observe any HBV reactivation [11,12]. In contrast, a retrospective analysis of patients treated with ICI showed reactivation rates ranging from 0.2% to 5.3% [13,14].

In studies that reported reactivation, a lack of antiviral prophylaxis was often a key factor. For instance, the rate of reactivation reached as high as 17.2% among chronic HBV patients who were not receiving antiviral prophylaxis, compared to just 1.2% in those who were on prophylaxis [14]. Based on available data, the strongest predictor of reactivation risk appears to be the absence of antiviral prophylaxis, followed by detectable baseline HBV DNA and HBsAg-positive (vs. isolated anti-HBc-positive) serologic status; cirrhosis and concurrent immunosuppressive therapy may further increase risk, though data remain limited. Most reported cases, as with our patient, were treated with either tenofovir or entecavir on diagnosis of HBV reactivation hepatitis and had a drop in HBV viral load, had normalization of liver enzymes and were able to be restarted on ICI with continued response with regard to cancer [5,6]. There was a case report in which treatment was not modified, with improvement in HBV DNA spontaneously on continued treatment with ICI and entecavir, but that patient was on entecavir prophylaxis prior to the rise in HBV DNA level.[10] Antiviral prophylaxis has been associated with a significantly lower risk of HBV reactivation in patients undergoing immunosuppressive or anticancer therapy [15]. Current evidence suggests that nucleos(t)ide analog therapy effectively suppresses HBV replication and reduces the risk of hepatitis flares during cancer treatment [16].

Based on our case report and a review of the literature, ICIs appear to be both effective and relatively safe for use in patients with chronic HBV infection. Patients who have reactivation of HBV can be successfully treated with antiviral therapy. The risk of HBV reactivation is notably lower in patients receiving such prophylaxis. Our patient was not placed on hepatitis B treatment prior to initiation of immunotherapy with nivolumab as there were no clear guidelines for management in patients undergoing immunotherapy that have been treated for hepatitis B in the past. Our patient was reportedly treated in Vietnam and had normal LFTs prior to diagnosis of cancer and initiation of treatment so we had no suspicion that the hepatitis B was active. There is a pressing need for updated HBV management guidelines to guide the close monitoring of these patients, including recommendations for use of antiviral prophylaxis in patients with history of HBV and chronic HBV cases, and guidelines on the frequency of HBV DNA titer monitoring during ICI therapy, duration of treatment, and target HBV DNA titers.

Current clinical guidelines recommend baseline screening with HBsAg, anti-HBc, and HBV DNA prior to initiation of immunosuppressive or anticancer therapies [16]. Periodic monitoring of HBV DNA and liver function tests during therapy allows for early detection and management of reactivation. HBV reactivation and hepatitis flare can lead to treatment interruptions, delays, or even the discontinuation of therapy, which may otherwise be avoidable with proper management. Prophylactic treatment is recommended for patients receiving treatments that are considered at high risk of hepatitis B reactivation such as anti-CD20 monoclonal antibodies, immunosuppressive systemic anticancer treatments or patients undergoing stem cell transplant [17]. However, whether patients receiving ICI need to be on prophylaxis is not made clear. The incidence of HBV reactivation with patients on ICI is low, and there is no consensus on whether to initiate patients with chronic HBV infection to be monitored or placed on prophylaxis [18].

An important clinical feature of this case is that nivolumab was successfully resumed after antiviral therapy, without recurrent transaminitis or worsening HBV-associated hepatitis. This suggests that HBV reactivation does not necessarily require permanent discontinuation of effective immunotherapy when promptly recognized and appropriately managed. For patients deriving substantial oncologic benefit from ICIs, early antiviral intervention and close hepatology–oncology collaboration may allow for safe treatment continuation or rechallenge. The limitation in our case is that this is a single case. We do not believe the one cycle of FOLFOX played a role as she had reactivation while she was off entecavir and was not on FOLFOX at that time. Prospective studies are needed to better define the risk of HBV reactivation across different ICIs and patient populations. Such data are essential to guide evidence-based management of viral hepatitis in patients receiving immunotherapy.

## 4. Conclusions

Patients receiving nivolumab remain at risk for hepatitis B reactivation, although this risk appears to be lower in those receiving antiviral prophylaxis. Currently, there are recommendations for HBV prophylaxis in patients receiving immunosuppressive anticancer treatments and anti-CD20 monoclonal antibodies; however, there is a lack of consensus guidelines for the prevention and management of HBV reactivation in patients treated with ICIs. In patients with a known history of HBV, regular monitoring of viral load may facilitate early detection and timely intervention. Given the potential for delayed reactivation after antiviral interruption, as demonstrated in this case, continued HBV DNA monitoring every 3–6 months for at least 6–12 months after ICI discontinuation is also advisable. Further prospective studies are needed to better define the risk of viral hepatitis reactivation across different ICIs and to inform evidence-based management strategies in this patient population.

## Figures and Tables

**Table 1:** Laboratory values before and after entecavir treatment.

Labs	At the Onset of Hepatitis B Flare	2 Weeks After Entecavir Treatment	2 Months After Entecavir Treatment
AST (U/L)	596	191	40
ALT (U/L)	480	246	44
ALP (U/L)	240	209	156
HBV DNA PCR (IU/mL)	6,894,517	25,900	1190
Bilirubin (mg/dL)	0.9	0.6	0.5
Hemoglobin (g/dL)	10.8	11.2	11.7
WBC (cells/µL)	3600	3900	4490
ANC (cells/µL)	1900	2100	2750
ALC (cells/µL)	1400	1100	1270
Platelet (platelet/µL)	106,000	130,000	114,000

Abbreviations: ALC: Absolute Lymphocyte Count; ALP: Alkaline Phosphatase; ALT: Alanine Amino Transferase; ANC: Absolute Neutrophil Count; AST: Aspartate Transferase; WBC: White Blood Cell.

## Data Availability

The original contributions presented in this study are included in the article. Further inquiries can be directed to the corresponding authors.
